# Same-Day CD4 Testing to Improve Uptake of HIV Care and Treatment in South Africa: Point-of-Care Is Not Enough

**DOI:** 10.1155/2013/941493

**Published:** 2013-07-16

**Authors:** Bruce A. Larson, Kathryn Schnippel, Alana Brennan, Lawrence Long, Thembi Xulu, Thapelo Maotoe, Sydney Rosen, Ian Sanne, Matthew P. Fox

**Affiliations:** ^1^Boston University Center for Global Health and Development, Boston University, Boston, MA 02118, USA; ^2^Department of International Health, Boston University School of Public Health, Boston University, Boston, MA 02118, USA; ^3^Health Economics and Epidemiology Research Office, Department of Clinical Medicine, School of Internal Medicine, Faculty of Health Sciences, University of the Witwatersrand, Johannesburg 2198, South Africa; ^4^Right to Care, Johannesburg 2041, South Africa; ^5^United States Agency for International Development, South Africa Mission, Pretoria 0027, South Africa; ^6^Department of Epidemiology, Boston University School of Public Health, Boston University, Boston, MA 02118, USA

## Abstract

*Background*. We evaluated whether a pilot program providing point-of-care (POC), but not rapid, CD4 testing (BD FACSCount) immediately after testing HIV-positive improved retention in care. *Methods*. We conducted a retrospective record review at the Themba Lethu Clinic in Johannesburg, South Africa. We compared all walk-in patients testing HIV-positive during February, July 2010 (pilot POC period) to patients testing positive during January 2008–February 2009 (baseline period). The outcome for those with a ≤250 cells/mm^3^ when testing HIV-positive was initiating ART <16 weeks after HIV testing. *Results*. 771 patients had CD4 results from the day of HIV testing (421 pilots, 350 baselines). ART initiation within 16 weeks was 49% in the pilot period and 46% in the baseline period. While all 421 patients during the pilot period should have been offered the POC test, patient records indicate that only 73% of them were actually offered it, and among these patients only 63% accepted the offer. *Conclusions*. Offering CD4 testing using a point-of-care, but not rapid, technology and without other health system changes had minor impacts on the uptake of HIV care and treatment. Point-of-care technologies alone may not be enough to improve linkage to care and treatment after HIV testing.

## 1. Introduction

A growing body of studies from resource limited settings documents poor patient retention in HIV care after HIV counseling and testing (HCT) [1]. Although comparing retention in preantiretroviral care across sites and countries is complicated by varying definitions and methods of measurement [2], it is clear that a large percentage of people testing HIV-positive at HCT sites do not return to collect CD4 test results, do not return on schedule for pre-ART monitoring and care, and/or do not initiate ART as soon as they become eligible [1, 3–6]. At Themba Lethu Clinic, which is a public-sector HIV/AIDS treatment facility at the Helen Joseph Hospital in Johannesburg, South Africa, for example, a retrospective review of patient records demonstrated that 65% of HIV-positive walk-in patients to the HCT program did not return for their CD4 test results within 12 weeks [3]. Among this cohort, nearly two-thirds (64%) of them were already eligible for ART on the day of HCT, based on having a CD4 count ≤ 200 cells/mm^3^

Because so many patients do not return for their CD4 count results, one improvement to the existing HCT practices that may reduce losses to HIV care and treatment after diagnosis is the use of point-of-care (POC) CD4 testing immediately after HCT [1, 6–10]. This strategy has the potential to improve the information available to both patients and providers and to reduce the number of visits required of patients, who would no longer have to make a second clinic visit just to receive test results. It may thus increase enrollment into pre-ART care or ART among patients who might otherwise never have returned to the clinic after their HIV test. On the other hand, it is also possible that POC CD4 testing will simply shift the problem of loss to care forward, with more patients receiving CD4 count results but with no change in uptake of care or treatment. In that case, the switch to a more expensive technology will not solve the problem it was designed to address.

Between February and July of 2010, the Themba Lethu Clinic piloted the introduction of POC CD4 testing in its HCT program using the BD FACSCount system. By offering same-day CD4 test results, combined with post-CD4 test counseling, the pilot program aimed to improve linkage to HIV care and treatment after HCT. We used retrospective data extracted from program records to evaluate whether this pilot program was successful in achieving this goal.

## 2. Methods

### 2.1. Study Setting

Themba Lethu HIV Clinic was founded in 2004 as part of the scaleup of HIV treatment [11]. The clinic, located in a public, academic hospital in the city of Johannesburg, is a comprehensive care, management, and treatment (CCMT) site that also receives support from a South African NGO called Right to Care, which in turn receives funding through the United States Agency for International Development under PEPFAR. The clinic offers HCT to walk-in patients and patients admitted to the hospital, performing more than 10,000 tests per year. By 2012, the site had initiated over 21,000 patients onto ART, making it one of the largest HIV clinics in South Africa [11]. 

Throughout the study periods and until July 2011, adults with a CD4 cell count ≤ 200 cells/mm^3^ were eligible for ART [12]. This policy was revised in August of 2012 to increase the CD4 cell count criterion to ≤ 350 cells/mm^3^.

### 2.2. Standard of Care

Under standard care at Themba Lethu Clinic, HCT patients receive pre- and posttest counseling that emphasizes the importance of CD4 testing for understanding disease progression and determining eligibility for antiretroviral therapy. Patients who test HIV-positive are encouraged to initiate standard CD4 testing immediately after posttest counseling by having a venipuncture blood draw by a nurse in a dedicated phlebotomy room in the clinic. The blood sample is sent to the National Health Laboratory Service (NHLS), where the test is run using the single platform panLeucogated CD4 platform [13]. The NHLS test results are typically made available to the site electronically within one week of HCT through the NHLS data system. All patients are counseled to return to the clinic after one week to obtain CD4 count results. 

At the next visit to the clinic, the CD4 test result is reviewed with the patient. During the study period, patients who had a CD4 count ≤ 250 cells/mm^3^ were “tracked for ART initiation,” which typically involved 2-3 additional visits to the site for medical assessment, education, and counseling prior to ART initiation [12]. Patients not eligible or tracked for ART initiation were enrolled in the clinic's wellness program. Those with a CD4 count between 250 and 350 were scheduled to return to the clinic after 3 months for a further medical exam and a new CD4 count, while those who had a CD4 count > 350 were scheduled to return after 6 months for the same services. 

### 2.3. Pilot Period

At the start of the pilot period, a CD4 flow cytometry machine, the BD FACSCount, was placed in the phlebotomy room, allowing CD4 counts to be conducted at the clinic rather than sending samples to the laboratory. A site phlebotomist was trained in using the machine. 

During the pilot period, POC CD4 testing was integrated into the routine cascade of care. During the pilot period, the intention was to offer the POC test to all patients testing HIV positive during HCT. During the pilot, patients received the same pre- and post-HIV-test counseling conveying the importance of CD4 testing as during the baseline period, but counselors also explained that the site could complete the CD4 test and provide results during the same visit. Patients consenting to the POC test had two vials of blood drawn from the same venipuncture; one was sent to the NHLS, while the phlebotomist tested the other one in the clinic using the on-site flow cytometry technology. Patients not opting for the POC test had one vial of blood drawn for NHLS testing.

Patients who opted for the POC CD4 test were then asked to wait in the clinic until the test was completed. Although a randomized clinical trial using the same technology reported that patients received test results within 45 minutes [6], during the pilot at TLC, the wait usually extended to 2-3 hours for a variety of reasons, including patient flow to the phlebotomist and waiting for more than one patient prior to running the test to allow for batch processing. After the test was complete, a counselor located the patient in the clinic, presented and discussed the POC CD4 test results with the patient, and explained whether the patient would be initially tracked for ART initiation or the wellness program. POC test results were recorded in the HCT logbook kept by the phlebotomist. No other changes were made to the HCT process during the pilot period, or in response to the availability of same-day CD4 tests.

During the pilot, consistent with the standard of care at the site, all patients were counseled to return to the site after one week. Patients who already received POC CD4 count results and were tracked for ART initiation could schedule this second visit to be the next sequential visit in the treatment initiation process (medical exam, additional laboratory tests, psychological assessment, adherence counseling, and so on). Patients who had already received a POC CD4 count result, but were tracked for the wellness program, received additional counseling on the importance of returning on schedule for their next pre-ART care visit 3 or 6 months later. At this second visit, the tracking decision for further medical care (ART or pre-ART care) could be confirmed for those patients completing the POC test or determined for those who did not, based on the NHLS CD4 result.

### 2.4. Data Collection and Analysis

We extracted data from clinic records for all walk-in HCT patients testing HIV-positive during a baseline (prepilot) period (January 2008 to February 2009) and during the pilot period (February 2010–July 2010) and for up to 8 months of followup. The data for the baseline period were published previously [3, 4]. Data for the pilot period included all walk-in HCT patients regardless of whether they accepted POC CD4 testing. We estimated the proportion of patients in each group (baseline and pilot) who initiated ART within 16 weeks of HIV testing (if CD4 ≤ 250) or completed their first wellness program visit within 4 weeks of a standard schedule (within 20 weeks of HCT if CD4 > 250 and CD4 ≤ 350, or within 32 weeks of HCT if CD4 > 350). For patients with CD4 ≤ 250 and tracked for ART initiation, 16 weeks allows for up to 4 weeks to return for CD4 results and then an additional 12 weeks to initiate ART. For patients with CD4 > 250 and CD4 ≤ 350, 20 weeks allows for up to 4 weeks to return for CD4 results, 12 weeks for the next return visit, and then an additional 4 weeks. For patients with CD4 > 350, 32 weeks allows for up to 4 weeks to return for CD4 results, 24 weeks for the next return visit, and then an additional 4 weeks.

Statistical analysis was conducted using STATA (version 11). A two-sample test of equality of proportions was used to test for differences between the two study periods. We present crude and adjusted risk differences (RDs) and relative risks (RRs) and corresponding 95% confidence intervals. Adjusted relative risks were estimated using a modified Poisson approach [14]. Models were adjusted for age, sex, and employment status. 

Ethical approval for this retrospective evaluation was obtained from Boston University and the University of the Witwatersrand.

## 3. Results

**Figure 1:**
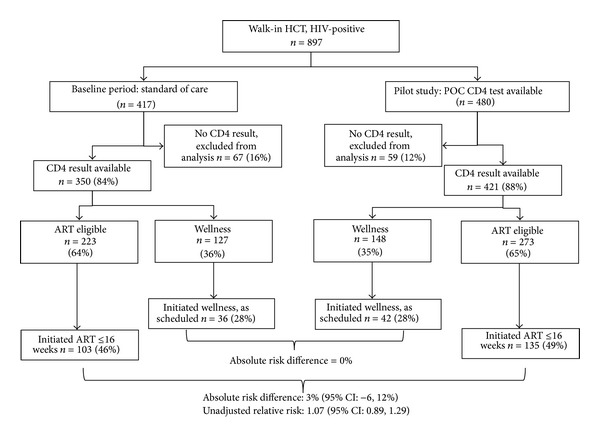
Summary of study groups, sample sizes, and outcomes.

Figure 1 provides an overview of the study. During the pilot and baseline periods, similar numbers (480 versus 417, resp.) of walk-in patients tested HIV-positive during HCT at the clinic. Although the site routinely requests a blood sample for CD4 testing as part of post-HIV-test counseling, not all patients provide a sample. A CD4 test result could not be identified either in patient records or in the NHLS database for 59 patients (12%) during the pilot period and 67 patients (16%) during the baseline period. These patients are excluded from further analysis.

Table 1 reports demographic and clinical characteristics by study group (baseline and pilot). Median age between the two groups is similar at about 37 years. The pilot group included fewer women (57% versus 64%) and employed patients (38% versus 50%) than the baseline group. Median CD4 cell counts on the day of HIV testing were very similar between the groups (166 and 177 cells/mm^3^ in the pilot and baseline groups, resp.).

### 3.1. Patients with CD4 Result ≤ 250 cells/mm^3^ (Tracked for ART Initiation)

As Figure 1 and Table 1 indicate, 65% of patients in the pilot group and 64% in the baseline group had a CD4 count below 250 cells/mm^3^ on the day of HCT and should have been tracked for ART initiation at the site. Only 46% and 49% of these patients initiated ART within 16 weeks during the baseline and pilot periods, respectively. Comparing the two groups, there was little absolute (unadjusted RR: 0.03, 95% CI: −0.06–0.12) or relative increase (unadjusted RR: 1.07, 95% CI: 0.89–1.29) in the proportion of eligible patients initiating ART within 16 weeks of testing positive. 

Relative risks adjusted for sex, employment status, and age are reported in Table 2. Regardless of group, employed individuals (compared to unemployed), women (compared to men), and younger (18–29 year olds) and older (50+ years) patients (compared to 30–39 year olds) were more likely to initiate ART within 16 weeks.

### 3.2. Patients with CD4 Result > 250 cells/mm^3^ (Tracked to the Wellness Program)

Of those patients with CD4 > 250 who were scheduled to return for a medical visit as part of the wellness program (see Figure 1), only 28% of patients in either study period returned within 4 weeks of the standard schedule for the wellness program. As reported in Table 3, relative risks of returning to the wellness program were substantially higher for employed patients (RR: 1.95, 95% CI: 1.32–2.87) and substantially lower for patients in the higher CD4 category (RR: 0.69; 95% CI: 0.47–1.00).

### 3.3. Implementation Issues during the Pilot Period

The results reported in Figure 1 and Tables 2 and 3 compare outcomes during the baseline period to outcomes for patients during the pilot period, regardless of whether patients in the pilot period actually received the intervention. During the implementation period, two practical issues arose that are relevant for other programs considering the introduction of POC CD4 testing after HCT to improve linkage to care. First, during the pilot program, while all 421 patients included in this analysis should have been offered the POC test, patient records indicate that only 308 (73%) were actually offered it. The exact reasons for this are not fully clear but seem to have been related to staffing levels, patient flow, and maintaining adequate supplies. 

If only the subset of patients offered the POC test during the pilot period is included in the analysis, 54% of those with a CD4 result ≤ 250 initiated ART within 16 weeks of HIV testing compared to 43% in the baseline period (unadjusted RD: 0.08; 95% CI: −0.01–0.16). Although the 54% in the intervention period for those offered the test represents an improvement upon the 49% observed in Figure 1 for all patients during the pilot period, an additional analysis suggests that women and employed patients were more likely to be offered the POC test. Thus, it is not clear if the improvement from 49% (all patients during pilot) to 54% (just patients offered the test) is related to being offered the test alone or that staff offered the test, whether intentionally or not, to patients who were already more likely to initiate ART. 

Second, among the 308 patients offered the POC test during the pilot period, only 63% (*N* = 194) accepted the offer to complete the POC test. It is likely that patients walking in to a clinic for HCT were simply unwilling to wait the extra 2-3 hours for their test results and therefore declined the offer. These same people might also be least likely to return for their CD4 test results a week later and thus remain in care. Women were more likely to accept the test than men, with little difference in acceptance between those employed or not.

Among the subset of patients who were offered the POC test, accepted the opportunity, and had a baseline CD4 result ≤ 250 (*N* = 110), 59% initiated ART within 16 weeks of HCT. The 59% observed in this subset of patients is substantially better than the 49% and 46% observed for all patients in the pilot and baseline periods. Because a substantial number of patients refused the offer of the POC test, these results very likely overestimate the impact of this intervention on linkage to HIV care and treatment in the general population. 

## 4. Discussion

Existing research and program experience shows that truly rapid point-of-care CD4 testing, such as with the Pima Analyzer (Alere), which produces a CD4 count in about 20 minutes, can readily be integrated into HIV counseling and testing programs and can have the potential to increase uptake of HIV care and treatment [6, 7, 15]. Given poor rates of linkage to care and the very low starting CD4 counts reported by cohort studies throughout Africa [1, 16], identifying even modestly effective ways to achieve this goal is potentially important.

Although there have been some successful demonstrations of point of care technologies, however, the benefits and costs of adopting these innovations are far from clear and are likely to vary widely by context and approach. We found that a less-rapid point of care technology, while feasible to implement and still creating the opportunity for same-day results, had little effect on patient behavior after a positive HIV test. The results presented here, based on patients presenting for HIV testing at a large, urban clinic, suggest that CD4 testing technology that is point of care but not rapid and that is not supported by other changes to clinic procedures has at best a minor impact on returning to care and initiating HIV treatment. In the pilot we evaluated that a majority of patients tracked for ART initiation on the day of testing HIV-positive did not initiate ART at the site with 16 weeks of HIV testing (52% across both study groups combined) and there was little improvement from the baseline period to the pilot period. Few patients with higher CD4 cell counts (>250) in each study period returned to the site for a next pre-ART care visit within one month of the recommended schedule; the point of care technology made no difference to this outcome.

As noted above, results presented in other studies are also modest. The largest improvement was reported in a randomized clinical trial at a nearby public hospital in Johannesburg, where patients were randomized to one of three groups (standard of care, standard of care with information, standard of care with information, and a POC CD4 test using the BD FACSCount). In this trial, 65% of patients receiving the POC test after HCT and found to be eligible for ART “reported for ART initiation” at a nearby ART clinic within 3 months of HIV testing. Compared to 31% of similar patients reporting in the standard of care arm, the study found a substantial improvement in this outcome (from 31% to 65%), although reporting for ART initiation is not the same as actually initiating ART. If such results held at scale this could indeed be an important benefit. However, given the very small sample size (only 43 patients receiving the POC CD4 test were ART eligible) and the trial conditions, under which patients consented to participate in the study and were then randomized to a study group, it is unclear to what degree such gains would be realized under routine conditions. It seems likely that under routine conditions, the trial site would experience many of the same operational constraints as did our evaluation site.

Other studies evaluating the potential impacts of providing CD4 test results more quickly to patients after HIV testing, whether using rapid POC technologies such as the Pima Analyzer or simply calling back patients with their CD4 results, have found modest improvements in linkage to care in fixed and mobile HCT programs [7, 15, 17]. Whether these improvements are “worth it”—whether the benefits in patient outcomes exceed the costs of procuring and utilizing more expensive technologies, and under what conditions—has not yet been established. What is clear is that the details matter. We found that simply offering point of care CD4 testing, using a technology that is same-day but not truly rapid, is not sufficient to achieve meaningful improvements in the health outcomes achieved.

Two tentative conclusions emerge from this evaluation. First, simple technological fixes might not be sufficient to address problems stemming from human behavior. Enrolling in care or treatment after HIV testing is fundamentally driven by patient-level behaviors and decisions, which are influenced by the information and services provided by the site, but not decided by the site. After an HIV diagnosis, some patients may not be able to focus on the implications of a specific CD4 count result, while other patients might very welcome the additional information. Even leaving HCT with clear knowledge of their eligibility for treatment and every intention to return on time to the site (their long-term preference), some patients may not actually make the decision to return on or near the scheduled day, for a range of emotional, social, and economic reasons (an example of short-term preferences at odds with long-term preferences). Offering better information to patients is important, but it is not a complete solution.

Second, new technologies may not be effective without health systems adaptations to accommodate and integrate them. In the case of the Themba Lethu Clinic pilot, the desire to process samples in batches meant that patients who arrived for HCT sporadically over the course of the day had to wait significantly longer than might otherwise have been necessary to receive their results. Performing HIV tests only during specified hours, rather than throughout the day, might have minimized this problem, though possibly at the expense of testing access. Similarly, shortages of supplies and staff meant that many patients were not offered POC testing at all, despite the presence of the POC instrument on site. In both instances, health systems changes were needed to support effective use of a new technology. 

Finally, it is important to note that in our study, a majority of walk-in HCT patients were already eligible for ART at the time of HIV diagnosis even under the restrictive threshold of 200 CD4 cells/mm^3^ that prevailed at the time of the study. Under the revised CD4 count eligibility criterion of 350, adopted in August 2011, some 80% of patients during the pilot period would have been eligible for ART at the time of their HIV test. The late disease stage at which most HIV-positive South Africans continue to learn of their infection and come forward for care underscores the importance of getting our linkage-to-care interventions right, with and without the benefits of new technologies. 

## Figures and Tables

**Table 1:** Study sample demographic and clinical characteristics at the time of testing HIV-positive*.

Characteristic	Baseline period		Pilot period	
	*N*	Result	*N*	Result
Age (median, IQR)	350	37 (32–44)	418	36 (31–44)
Female (proportion)	348	64%	421	57%
Employed (proportion)	350	50%	421	38%
CD4 (median, IQR)	350	177 (63–331)	421	166 (72–314)
Eligible for ART or ART tracking (%)		64%		65%

*These are patients with a CD4 test result available from the same date as HIV testing. The NHLS result is used for all patients in the baseline group and all but 10 patients in the pilot period, where the Pima result is used because an NHLS result could not be found in patient records or the NHLS database.

**Table 2:** Adjusted relative risks of initiating antiretroviral therapy within 16 weeks of HIV counseling and testing for patients with CD4 count ≤ 250 on the day testing HIV-positive.

Variable	Adjusted relative risks (95% CI)*	*P* value
Pilot period	1.20 (0.99–1.46)	0.06
Baseline period	Reference	
Employed	1.45 (1.20–1.76)	0.00
Unemployed	Reference	
Female	1.24 (1.02–1.51)	0.03
Male	Reference	
Age 18–29	1.29 (1.00–1.65)	0.05
Age 30–39	Reference	
Age 40–49	1.13 (0.90–1.44)	0.30
Age 50+	1.32 (1.00–1.75)	0.05

*Adjusted relative risks estimated using modified Poisson approach.

**Table 3:** Relative risks of returning to wellness program for patients with CD4 > 250 on the day of testing HIV-positive.

Variable	Adjusted relative risks (95% CI)	*P* value
Pilot Period	1.02 (0.69–1.50)	0.92
Baseline period	Reference	
Employed	1.95 (1.32–2.87)	0.00
Unemployed	Reference	
Female	1.16 (0.77–1.75)	0.47
Male	Reference	
Age 18–29	0.50 (0.28–0.91)	0.02
Age 30–39		*
Age 40–49	0.71 (0.42–1.19)	0.19
Age 50+	1.27 (0.77–2.09)	0.34
CD4 ≤ 350 on day of HIV testing	Reference	*
CD4 > 350 on day of HIV testing	0.68 (0.47–1.00)	0.05

*Relative risks estimated using modified Poisson approach.
